# Are the mediolateral joint forces in the lower limbs different between scoliotic and healthy subjects during gait?

**DOI:** 10.1186/1748-7161-10-S2-S3

**Published:** 2015-02-11

**Authors:** Mouna Yazji, Maxime Raison, Carl-Éric Aubin, Hubert Labelle, Christine Detrembleur, Philippe Mahaudens, Marilyne Mousny

**Affiliations:** 1 Polytechnique Montréal, Montreal, Canada; 2 Research Center of Sainte-Justine UHC, Montreal, Canada; 3 Institute of Neurosciences (IoNS), Université Catholique de Louvain (UCL), Bruxelles, Belgium; 4 Service d'Orthopédie et de Traumatologie de l’appareil locomoteur (ORTO), Saint-Luc UHC, Brussels, Belgium

**Keywords:** Idiopathic scoliosis, Gait, Hip, Medio-lateral force, Inverse dynamics, Human body

## Abstract

**Introduction:**

The quantification of internal joint efforts could be essential in the development of rehabilitation tools for patients with musculo-skeletal pathologies, such as scoliosis. In this context, the aim of this study was to compare the hips joint mediolateral forces during gait, between healthy subjects and adolescents with left lumbar or thoracolumbar scoliosis (AIS), categorized by their Cobb angle (CA).

**Material and methods:**

Twelve healthy subjects, 12 AIS with CA between 20° and 40° and 16 AIS in pre-operative condition (CA : > 40°) walked at 4 km/h on an instrumented treadmill. The experimental set-up include six infrared cameras allow the computation of the tridimensional (3D) angular displacement and strain gauges located under the motor-driven treadmill allow the computation of ground reaction forces (GRF). The hips joint mediolateral forces were calculated using a 3D inverse dynamic of human body. One-way ANOVA was performed for the maximum, the minimum and the range of medio-lateral forces at each joint of the lower limbs. When appropriate, a Tukey's post hoc was performed to determine the differences.

**Results:**

The mediolateral forces were significantly lower at the right hip for AIS with CA between 20° and 40° compared to healthy subject.

**Conclusion:**

The spinal deformation leads to a reduced medio-lateral force at the right hip, which could gradually change the scheme of postural adjustments for AIS during gait. Further research on the quantification of the joint lower limb efforts should include the knee and ankle joints to evaluate the impact of spinal deformation on the lower limb dynamic behaviour in AIS patients.

## Introduction

Idiopathic scoliosis has been characterized by decreased stability and standing imbalance, which may result from the reduced muscle efficiency that results from the longer contraction time of the lumbar and pelvic muscles [1]. The quantification of medio-lateral force during gait could help to select specific postural rehabilitation exercises around each lower limb joint to develop indicators of quality and comfort during gait. In this context, the aim of this study was to compare the hips joint mediolateral forces during gait, between healthy subjects and left lumbar or thoracolumbar AIS with different scoliotic severities, categorized by their Cobb angle (CA).

## Material and methods

Twelve healthy subjects, 12 adolescents with idiopathic scoliosis (AIS) with Cobb angle (CA) between 20° and 40° and 16 AIS in pre-operative condition (Cobb : > 40°) performed gait at 4 km/h on an instrumented treadmill. All subjects were submitted to a neurological examination. Patients with neurological abnormalities observed on clinical examination were not included in this study [2]. The acquisition system [3] included optokinetic sensors (*BTS*, Italy) recording the 3D joint coordinates and a treadmill equipped with force sensors (*UCL*, Belgium) measuring the 3D external forces independently applied to each feet. The hip joint forces at the left and right lower limbs were calculated using a tridimensional inverse dynamic model of the human body.

**Figure 1:**
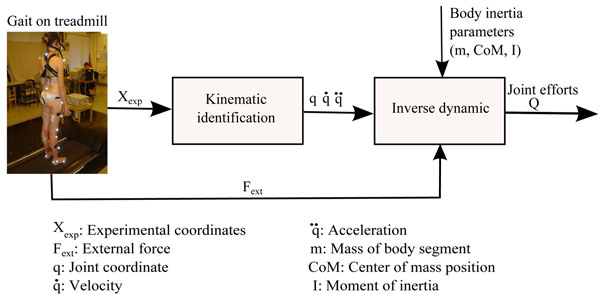
Principe of inverse dynamics to quantify the lower limbs efforts.

Using these data, a 3D multibody model of the human body provided hip joint medio-lateral forces, via these three consecutive steps (Figure 1):

1. Kinematic identification: From the raw kinematic data of the optokinetic sensors, we compute the joint center locations (Cartesian coordinates) of the pelvis, the hip, the knee and the ankle as proposed by Davis et al., 1991[4]. Then, from the Cartesian coordinates of the joint centers, the joint coordinates ***q***, are numerically determined by a global optimisation method (GOM) as proposed by Lu et al., 1999 [5] that estimates the joint coordinates of the multibody model that best fit the experimental joint centers positions [1]. The optimal pose of the multibody model is computed for each data frame such that the difference between the experimental and model-determined marker coordinates is globally minimized in a least squares sense. Finally, the corresponding velocities  and accelerations  are presently estimated from ***q*** using a numerical derivative using cubic splines.

2. Decomposition of the ground reaction forces: The decomposition of the total ground reaction forces (GRF) measured by the platform set-up is computed as proposed by Ballaz et al., 2013 [6] to obtain the GRF under each foot. We should mention that this algorithm is only approved on vertical component of GRF. However, the horizontal force is a small component of the total GRF and by consequence its error had only a small effect on joint moments as proposed by Stoquart et al., 2008 [7].

3. Inverse dynamics: the dynamical equations of the system are obtained from a Newton-Euler formalism [8]: this algorithm provides the vector ***Q*** of internal interaction torques and forces at the joints for any configuration of the multibody system, in the form of an inverse dynamical model (Equation 1) which is generated by *Robotran* software :

Normality of the distributions was determined using the Shapiro test. One-way ANOVA was performed for the maximum, the minimum and the range of medio-lateral forces at the hip joint of the lower limbs. When appropriate, a Tukey's post hoc was performed to determine the differences. *P* < 0.05 was used to define the significance threshold. Statistical analyses were performed using Statistica. Furthermore, the repeatability between right and left side in normal subjects was tested by using the interclass correlation coefficient (ICC) to make sure that the method of computation was accurate in establishing relevant and significant differences between right and left for AIS patient in a future study.

## Results

**Figure 2:**
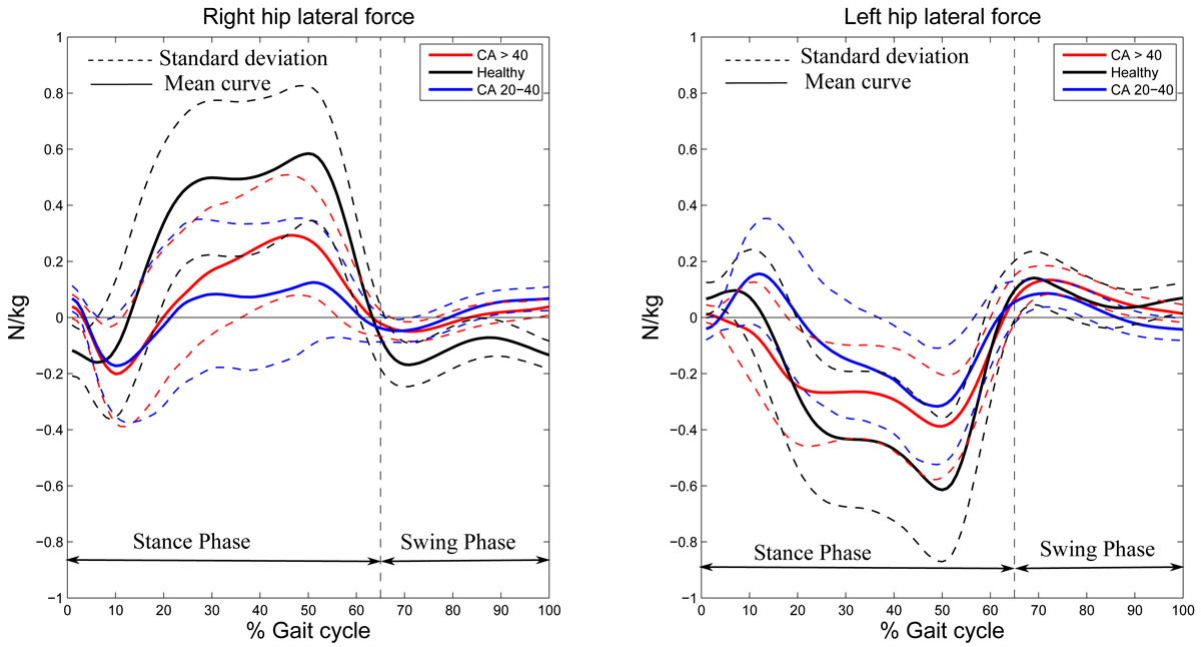
Mean values of hip joint mediolateral forces (N/kg) are presented as a function of gait cycle time (%).

The maxima (p-value = 0.04) and the magnitudes (p-value = 0.02) of the mediolateral forces at the right hips were found to be different across the groups (Figure 2). The Tukey multiple comparisons found that the right hips medio-lateral forces were significantly lower in AIS having a CA between 20° and 40° compared to healthy subjects. The left mediolateral forces showed no significant differences between healthy subjects and the two AIS subgroups.

## Discussion

The mediolateral joint forces at the right hip are significantly different between AIS and healthy subjects. The study does not indicate significant difference between the 2 AIS groups for the mediolateral forces at the hips joint. The spinal deformity correlates to a decrease of hip mediolateral forces. This force decrease can be explained by the reduced muscle efficiency that results from the longer contraction time of the lumbar and pelvic muscles. This force decrease could gradually change the pattern of postural adjustments in AIS during gait. Especially at the hip, the decreased mediolateral force corresponds to a postural adjustment balancing the increased pelvic moment generated by the mediolateral shift of the thoracic mass during gait.

The present study does not report significant differences between healthy subjects and AIS groups at the left hip mediolateral force. This finding could be explained by the fact that AIS patient have left lumbar or thoracolumbar curvature so the right side will exert less important forces to compensate the curvature on the left side.

Also, the present study does not show significant differences between healthy subjects and AIS in pre-operative condition (Cobb : > 40°). This surprising finding that more severe curves do not demonstrate a more pronounced difference than less severe curves could be related to an adaptive phenomenon over the long term as the stiffness in AIS patients is internal and permanent.

## Conclusion

For the first time, a study shows the interest of the quantification of the internal efforts as a functional assessment indicator for scoliosis during gait, as is already done for many pathologies. Consequently, the evaluation of the mediolateral forces in the lower limbs could help to select specific postural rehabilitation exercises around each lower limb joint to develop indicators of quality and comfort during gait.

For future studies, we suggested that subjects undergo a MRI scan beside the neurological examination to make sure that no patients are included in the study with subtle neuromuscular disorder.

Finally, these results open the perspective of a more extended study on the quantification of the lower limb joint efforts for the evaluation of the spine deformation on the knee and ankle joint in scoliotic patients during gait. The information provided by the quantification of internal joint efforts could be essential in the development of rehabilitation tools for patients with musculo-skeletal pathologies.

This is the extended abstract of IRSSD 2014 program book [9].

## Consent

Written informed consent was obtained from the patient for publication of this Case Report and any accompanying images. A copy of the written consent is available for review by the Editor-in-Chief of this journal.

Each subject signed on and participated freely in study, approved by the local ethics board at Université Catholique de Louvain (UCL) as mentioned in the study of Mahaudens et al., 2009 [1]. In fact, in this study, we have used the same data base of patients as the study of Mahaudens et al., 2009 [1].

## Abbreviations

AIS: Adolescent idiopathic scoliosis; CA: Cobb angle; 3D: Three dimensional; GRF: Ground reaction forces; cm: Centimeter; ICC: Interclass correlation coefficient

## Competing interests

The authors declare that they have no competing interests.

## Authors' contributions

MY participated in the development of 3D multibody model of the human body and the interpretation of data. MR helped to draft and to revise the manuscript. CD and PM performed the data collection. All authors read and approved the final manuscript.

## Declarations

Publication charges for this collection were funded by IRSSD 2014 Sapporo.

This article has been published as part of *Scoliosis* Volume 10 Supplement 2, 2015: Research into Spinal Deformities 9: Short Papers from the IRSSD 2014 Meeting. The full contents of the supplement are available online at http://www.scoliosisjournal.com/supplements/10/S2.
